# The role of lymphocyte-C-reactive protein ratio in the prognosis of gastrointestinal cancer: a systematic review and meta-analysis

**DOI:** 10.3389/fonc.2024.1407306

**Published:** 2024-08-29

**Authors:** XiaoMeng Liu, JingChen Zhang, HaoYu An, WanYao Wang, YuKun Zheng, FengJiang Wei

**Affiliations:** ^1^ School of Basic Medicine, Tianjin Medical University, Tianjin, China; ^2^ National Population Health Data Center, Chinese Academy of Medical Sciences & Peking Union Medical College, Beijing, China; ^3^ School of Medicine, The Chinese University of Hong Kong, Hong Kong, Hong Kong SAR, China; ^4^ School of Food Science and Engineering, Tianjin University of Science and Technology, Tianjin, China

**Keywords:** lymphocyte-C-reactive, prognosis, gastrointestinal cancer, meta-analysis, systematic review

## Abstract

**Objective:**

The lymphocyte-to-C-reactive protein (LCR) ratio, an immune-inflammatory marker, shows prognostic potential in various cancers. However, its utility in gastrointestinal malignancies remains uncertain due to inconsistent findings. This systematic review and meta-analysis synthesizes recent evidence to elucidate the association between LCR and prognosis in gastrointestinal cancer patients, aiming to clarify LCR’s potential role as a prognostic biomarker.

**Methods:**

We searched PubMed, Embase, Cochrane, and Web of Science databases up to May 2024 to evaluate the association between LCR and prognosis in gastrointestinal cancer patients. The main outcomes included overall survival (OS), recurrence-free survival (RFS), and disease-free survival (DFS). We also analyzed secondary parameters such as geographical region, study duration, sample size, LCR threshold, and patient characteristics (age, gender, tumor location, and TNM stage).

**Results:**

This meta-analysis of 21 cohort studies (n=9,131) finds a significant association between reduced LCR levels and poor prognosis in gastrointestinal cancer. Lower LCR levels were associated with worse overall survival (HR=2.01, 95% CI=1.75-2.31, *P*<0.001), recurrence-free survival (HR=1.90, 95% CI=1.32-2.76, *P*<0.001), and disease-free survival (HR=1.76, 95% CI=1.45-2.13, *P*<0.001). Subgroup analyses by cancer type, timing, and LCR threshold consistently confirmed this relationship (*P*<0.05).

**Conclusion:**

LCR may serve as a prognostic marker in gastrointestinal cancer patients, with lower LCR levels associated with poorer prognosis. However, more high-quality studies are needed to validate these findings, considering the limitations of the current evidence.

**Systematic review registration:**

https://www.crd.york.ac.uk/prospero/, identifier CRD42023486858.

## 1 Introduction

Gastrointestinal cancer, which includes esophageal cancer (EC), gastric cancer (GC), and colorectal cancer (CRC), is a major contributor to global cancer-related mortality. In 2020, gastrointestinal cancer accounted for 2,228,749 deaths worldwide (1). Among gastrointestinal cancers, CRC was the second most prevalent at 9.4%, followed by GC (7.7%) and EC (5.5%). The incidence of gastrointestinal cancers is expected to continue increasing over the next decade. In Asia, the incidence of GC is projected to reach approximately 20 cases per 100,000 individuals (2). Moreover, the global burden of GC is expected to increase by 62% by 2040 (3), while CRC incidence is projected to reach 3.2 million new cases, with 1.6 million deaths by 2040 (4). Despite advancements in multidisciplinary treatments, gastrointestinal cancer mortality remains high. Prognostic biomarkers are needed to identify high-risk patients and enable personalized therapy, potentially improving prognosis.

Cancer-associated inflammation is linked to intratumoral immunosuppression and cancer progression. Systemic inflammation is associated with reduced survival in various malignancies due to mucosal injury and DNA damage (5, 6). Numerous studies have shown that systemic inflammation predicts tumor recurrence and survival in various cancers, including gastrointestinal cancer. Parameters such as the preoperative neutrophil-to-lymphocyte ratio (NLR) (7), platelet-to-lymphocyte ratio (PLR) (8), Prognostic Index (PI) (9), Prognostic Nutritional Index (PNI) (10), Glasgow Prognostic Score (GPS) (11), and Modified Glasgow Prognostic Score (mGPS) (12) have been identified. CRP and lymphocyte levels are reliable indicators of post-operative infections and immune status. Lymphocytes have high specificity but low sensitivity, while CRP has low specificity but high sensitivity (13). Thus, lymphocyte-to-CRP ratio (LCR) may better reflect inflammatory status (14). Recent studies have highlighted LCR’s potential in gastrointestinal cancer (15–37). However, LCR’s role is not fully understood, and most meta-analyses have focused on specific cancers. This study conducts a comprehensive meta-analysis across gastrointestinal cancers to explore the correlation between LCR and prognosis.

## 2 Methods

### 2.1 Literature search

We conducted a comprehensive literature search of PubMed, Embase, Cochrane, and Web of Science databases up to May 2024 to identify English studies investigating the association between LCR and prognosis in gastrointestinal cancer. The search terms included: “lymphocyte”, “C-reactive protein”, “ratio”, “gastrointestinal cancer”, “colorectal cancer”, “gastric cancer”, and “esophageal cancer” (detailed search strategy in **Supplementary Table 1**). Reference lists of eligible studies were manually scrutinized. Two investigators (XML and JCZ) independently conducted the search and study selection, with discrepancies resolved through consensus. This study was preregistered in PROSPERO (CRD42023486858). Studies were included if they met the following criteria: P: patients diagnosed with gastrointestinal cancer; I: low LCR; C: high LCR; O: at least one survival outcome, such as overall survival (OS), disease-free survival (DFS), or recurrence-free survival (RFS); S: cohort studies. Exclusions comprised reviews, letters, editorials, case reports, conference abstracts, inadequate data, duplicate literature, and non-English articles.

### 2.2 Data extraction

Data extraction was conducted independently by two investigators (XML, JCZ), with any disparities resolved by a third investigator (HYA) to achieve consensus. The extracted information from the studies included essential details such as the first author, publication year, study period, country, design, cancer type, sample size, age, gender, body mass index (BMI), timing, tumor size, TNM stage, LCR threshold, and relevant outcome measures.

### 2.3 Quality evaluation

The Newcastle–Ottawa Scale (NOS) was employed to assess study quality, which including representativeness, selection of non-exposed, ascertainment of exposure, outcome not present at start, comparability on most important factors, comparability on other risk factors, assessment of outcome, long enough follow-up (median≥1 year) and adequacy (completeness) of follow-up. Studies scoring seven to nine points were considered high quality (38), as per the scale’s criteria. Two investigators independently evaluated the quality and level of evidence for eligible studies, and any discrepancy was resolved through discussion.

### 2.4 Statistical analysis

The study utilized Hazard Ratios (HR) with a 95% Confidence Interval to assess the correlation between Lymphocyte-to-CRP Ratio (LCR) and prognosis (OS, DFS, RFS, etc.) in gastrointestinal cancer patients. Subgroup analyses were conducted based on LCR thresholds, cancer types, and timing to explore its impact on prognosis. Heterogeneity was assessed using the *I* ^2^ statistic and Q test, considering significance at *P*<0.1 and/or *I* ^2^>50%. Meta-analysis employed a random-effects model for significant heterogeneity; otherwise, a fixed-effects model was used. Sensitivity analyses were performed for indicators with all included studies. Funnel plot and Egger’s test were used for outcomes that included all literature to evaluate publication bias with a *P*-value of 0.05. All statistical analyses were performed using Review Manager 5.4 versions and STATA 15.0.

## 3 Results

### 3.1 Literature search and study characteristics

**Figure 1:**
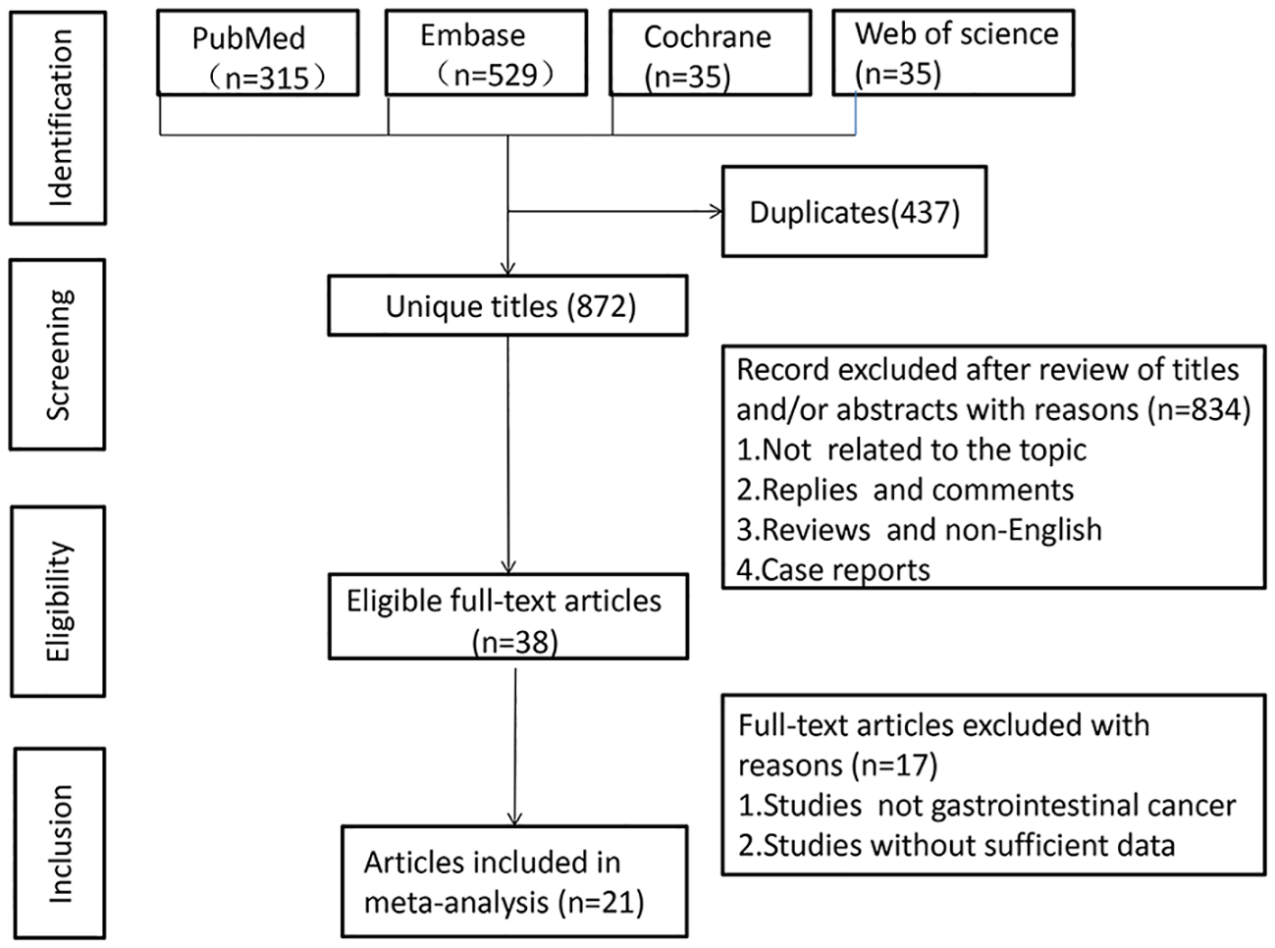
Flowchart of the systematic search and selection process.

A literature search across PubMed (n = 315), Embase (n = 529), Cochrane (n = 35), and Web of Science (n = 430) yielded 1,309 articles (**Figure 1**). After removing duplicates, 872 titles and abstracts were reviewed. Finally, 21 articles, involving 9,131 patients, were included. One study was a prospective cohort (23), while 20 were retrospective cohorts. The studies, published between 2019-2023, included 18 from Japan and 3 from China. Twenty studies examined the association between LCR and OS, six explored LCR and DFS, and eight investigated LCR and RFS. LCR was defined as lymphocytes-to-C-reactive protein ratio in 19 studies, while three used the CLR (28, 31, 32). **Table 1** summarizes the characteristics and quality scores (median: 9). **Supplementary Table 2** provides quality assessment details.

**Table 1:** Baseline characteristics of include studies and methodological assessment.

Authors	Study period	Country	Study design	Types of cancer	Timing	Patients	Gender Male/Female	Age	Treatment method	T stage	LCR threshold	Quality score
Aoyama et al. (13)	2013-2017	Japan	retrospective	gastric cancer	preoperative	480	318/162	68	surgical procedures and chemotherapy	T1-T4	7000	9
Yamamoto et al. (12)	2010-2018	Japan	retrospective	rectal cancer	preoperative	202	121/81	67	surgical procedures and chemotherapy or chemoradiotherapy	T0-T4	12600	7
Aoyama et al. (11)	2008-2018	Japan	retrospective	esophageal cancer	preoperative	89	77/12	68	surgical procedures and chemotherapy	T1-T3	12177	7
Yamamoto et al. (10)	2002-2017	Japan	retrospective	esophageal cancer	preoperative	153	128/25	69	surgical procedures and chemotherapy	T0-T4	7842	9
Tsujiura et al. (13)	2002-2020	Japan	retrospective	gastric cancer	preoperative	103	85/18	68	surgical procedures and chemotherapy	T1-T4	4610	8
Miyatani et al. (14)	2005-2018	Japan	retrospective	gastric cancer	preoperative and postoperative	455	332/123	75	surgical procedures	T1-T4		9
Okugawa et al. (14)	2006-2015	Japan	retrospective	colorectal cancer	preoperative and postoperative	307	183/124	68	surgical procedures	T1-T4	6676	9
Nishi et al. (11)	2004-2012	Japan	retrospective	rectal cancer	preoperative and postoperative	48	32/16	66	surgical procedures	T1-T3	11,765	9
Okugawa et al. (21)	2019	Japan	prospective	colorectal cancer	preoperative	477		71	surgical procedures	T1-T4	6000	8
Okugawa et al. (15)	2019	Japan	retrospective	gastric cancer	preoperative	551	387/164	65.3	surgical procedures	T1-T4		8
Takeuchi et al. (10)	2000-2019	Japan	retrospective	esophageal cancer	preoperative	495	421/74	65	surgical procedures and chemotherapy	T1-T4	19000	9
Nakamura et al. (8)	2000-2015	Japan	retrospective	colorectal cancer	preoperative	756	435/321	61	chemotherapy		889	9
Utsumi et al. (11)	2010-2021	Japan	retrospective	colorectal cancer	preoperative	104	63/41	66.7	surgical procedures and chemotherapy or chemoradiotherapy	T1-T4	12720	9
Kono et al. (11)	2003-2014	Japan	retrospective	gastric cancer	postoperative	227	166/61	68.8	surgical procedures and chemotherapy	T1-T4	152.6	8
Matsunaga et al. (13)	2017-2022	Japan	retrospective	gastric cancer	preoperative	101	79/22	65.1	chemotherapy			8
Okugawa et al. (14)	2001-2015	Japan	retrospective	rectal cancer	preoperative	86	64/22	64	surgical procedures and chemotherapy	T1-T4	6000	9
Meng et al. (7)	2004-2019	China	retrospective	colorectal cancer	preoperative	2471	971/1500	57.26	surgical procedures	T0-T4	0.2	8
Taniai et al. (8)	2000-2018	Japan	retrospective	colorectal cancer	preoperative	197	137/60	65	surgical procedures and chemotherapy or chemoradiotherapy		15900	9
Ou et al. (5)	2010-2014	China	retrospective	colorectal cancer	preoperative	955	540/415	65	surgical procedures and chemotherapy	T1-T4	6500	8
Cheng et al. (4)	2013-2019	China	retrospective	gastric cancer	postoperative	607	196/411	58.65	surgical procedures and chemotherapy	T1-T4	0.63	9
Sawada et al. (11)	2005-2014	Japan	retrospective	rectal cancer	preoperative	267	181/86	59.5	surgical procedures and chemotherapy or chemoradiotherapy	T2-T4	18141.4	9

### 3.2 Meta-analysis results

#### 3.2.1 The relationship between LCR and OS

**Figure 2:**
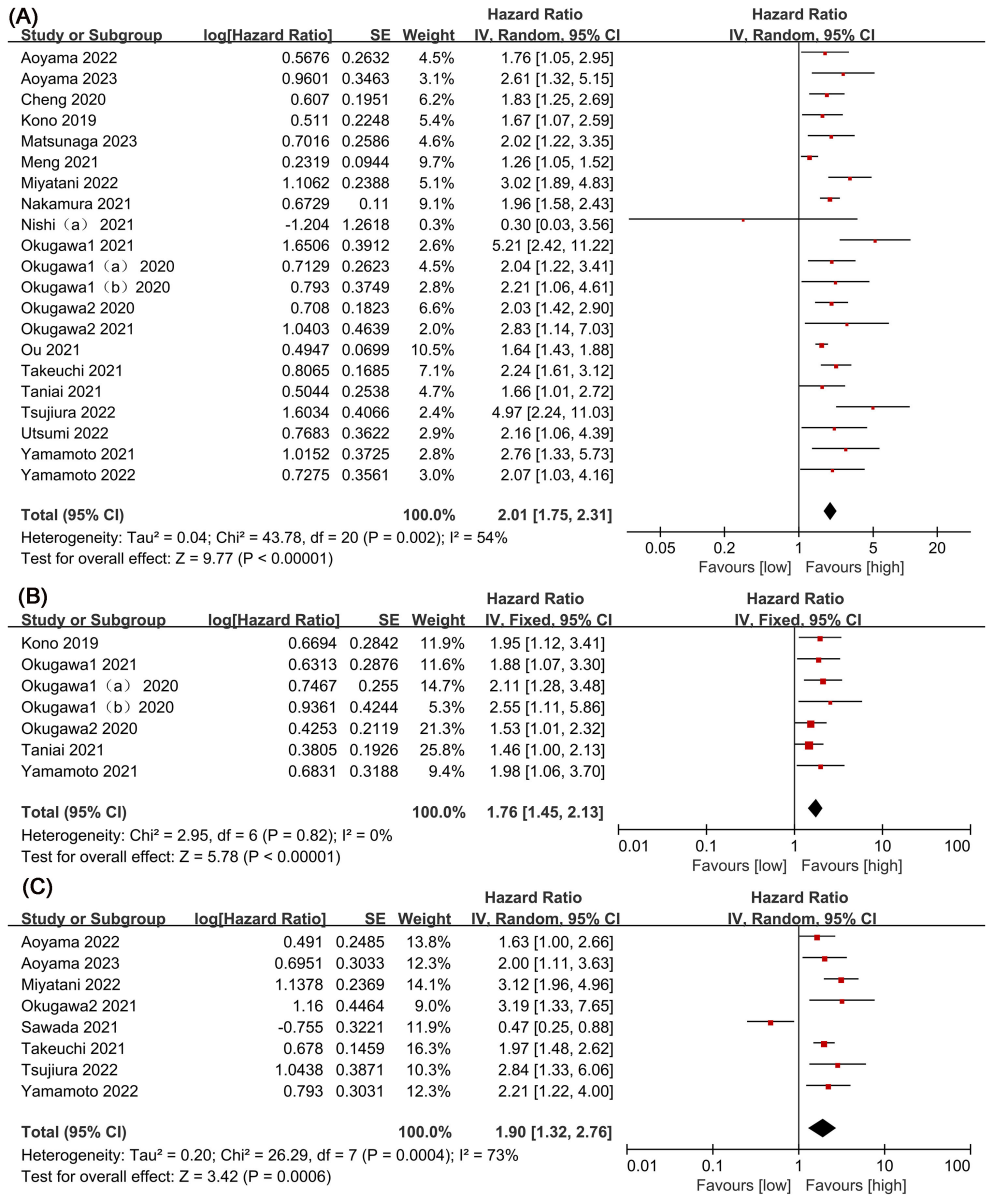
Forest plots of perioperative outcomes: **(A)** overall survival (OS), **(B)** disease-free survival (DFS), **(C)** recurrence-free survival (RFS).

The association between LCR and overall survival (OS) was investigated in 20 studies, revealing significant heterogeneity (*I* ^2^ = 54%, *P* = 0.002). Using a random-effects model, our meta-analysis showed that patients with lower LCR levels had significantly worse OS (HR = 2.01, 95% CI = 1.75-2.31, *P* < 0.00001) (**Figure 2A**). Subgroup analyses (**Table 2**), including different cancer types, timing, LCR thresholds, and treatment method, maintained predictive significance (*P* < 0.05). Heterogeneity decreased with preoperative (*I* ^2^ = 48%, *P* < 0.00001) and postoperative timing (*I* ^2^ = 0%, *P* = 0.00001), suggesting timing contributed to heterogeneity.

**Table 2:** Subgroup analysis of OS, RFS and DFS for gastrointestinal cancer.

Subgroup	OS				RFS				DFS			
	Study	HR [95%CI]	*P* value	*I* ^2^	Study	HR [95%CI]	*P* value	*I* ^2^	Study	HR [95%CI]	*P* value	*I* ^2^
***Total***	21	2.01[1.75-2.31]	<0.00001	54%	8	1.90[1.32-2.76]	0.0006	73%	7	1.76[1.45-2.13]	<0.00001	0%
*Types of cancer*												
gastric cancer	7	2.09[1.76-2.48]	<0.00001	32%	3	2.37[1.75-3.23]	<0.00001	47%	2	1.67[1.20-2.33]	0.003	0%
esophageal cancer	3	2.37[1.80-3.12]	<0.00001	0%	2	1.98[1.53-2.56]	<0.00001	0%	1	1.98[1.06-3.70]	0.03	NA
colorectal cancer	8	1.82[1.48-2.23]	<0.00001	67%	0				4	1.78[1.38-2.29]	<0.00001	0%
rectal cancer	3	2.11[1.23-3.62]	0.007	28%	3	1.46[0.46-4.67]	0.52	88%	0			
*Timing*												
preoperative	17	1.73[1.60-1.88]	<0.00001	48%	7	1.76[1.18-2.62]	0.006	72%	5	1.71[1.38-2.13]	<0.00001	0%
postoperative	2	1.76[1.32-2.35]	0.00001	0%	0				1	1.95[1.12-3.41]	0.02	NA
*LCR threshold*												
≤7000	11	1.92[1.59-2.32]	<0.00001	66%	3	2.10[1.45-3.05]	<0.0001	20%	4	2.05[1.53-2.74]	<0.00001	0%
>7000	7	2.13[1.72-2.65]	<0.00001	0%	4	1.46[0.78-2.71]	0.23	83%	2	1.59[1.15-2.19]	0.005	0%
*Treatment method*												
surgical procedures surgical procedures and chemotherapy surgical procedures and chemotherapy or chemoradiotherapy chemotherapy	7 9 3 2	2.13[1.43-3.18] 2.18[1.74-2.74] 1.87[1.32-2.66] 1.97[1.61-2.40]	0.0002 <0.00001 0.0005 <0.00001	78% 59% 0% 0%	1 5 2 0	3.12[1.96-4.96] 2.02[1.63-2.49] 1.02[0.22-4.66]	<0.00001 <0.00001 0.98	NA 0% 92%	4 2 1 0	1.84[1.42-2.40] 1.96[1.30-2.98] 1.46[1.00-2.13]	<0.00001 0.001 0.05	0% 0% NA

NA = not available.

#### 3.2.2 Relationship between LCR and DFS

Six studies were examined to assess the correlation between LCR and DFS, revealing no significant heterogeneity (*I* ^2^ = 0%, *P* = 0.82). Using a fixed-effect model, our meta-analysis demonstrated a strong association between lower LCR and worse DFS (HR = 1.76, 95% CI = 1.45-2.13, *P* < 0.00001) (**Figure 2B**). Subgroup analysis (**Table 2**) identified significant differences in cancer types, timing, LCR threshold and treatment method (*P* < 0.05).

#### 3.2.3 Relationship between LCR and RFS

Eight studies explored the association between LCR and RFS, unveiling notable heterogeneity (*I* ^2^= 73%, *P*=0.0004). Employing a random-effects model to address this heterogeneity, the meta-analysis revealed a significant correlation: lower LCR levels associated with markedly diminished RFS (HR=1.90, 95%CI=1.32-2.76, *P*=0.0006) (**Figure 2C**). Subgroup analysis (**Table 2**) indicated persistent significance in studies stratified by cancer type and timing (*P*<0.05), while significance diminished in rectal cancer studies (*P*=0.52), LCR threshold>7000 studies (*P*=0.23) and surgical procedures and chemotherapy or chemoradiotherapy studies (P=0.98). The potential prognostic value of LCR for rectal cancer and LCR threshold>7000 warrants further investigation. Notably, timing emerged as a major source of heterogeneity (*I* ^2^ = 72, *P*=0.006), suggesting its pivotal role in influencing study outcomes.

#### 3.2.4 Publication bias

**Figure 3:**
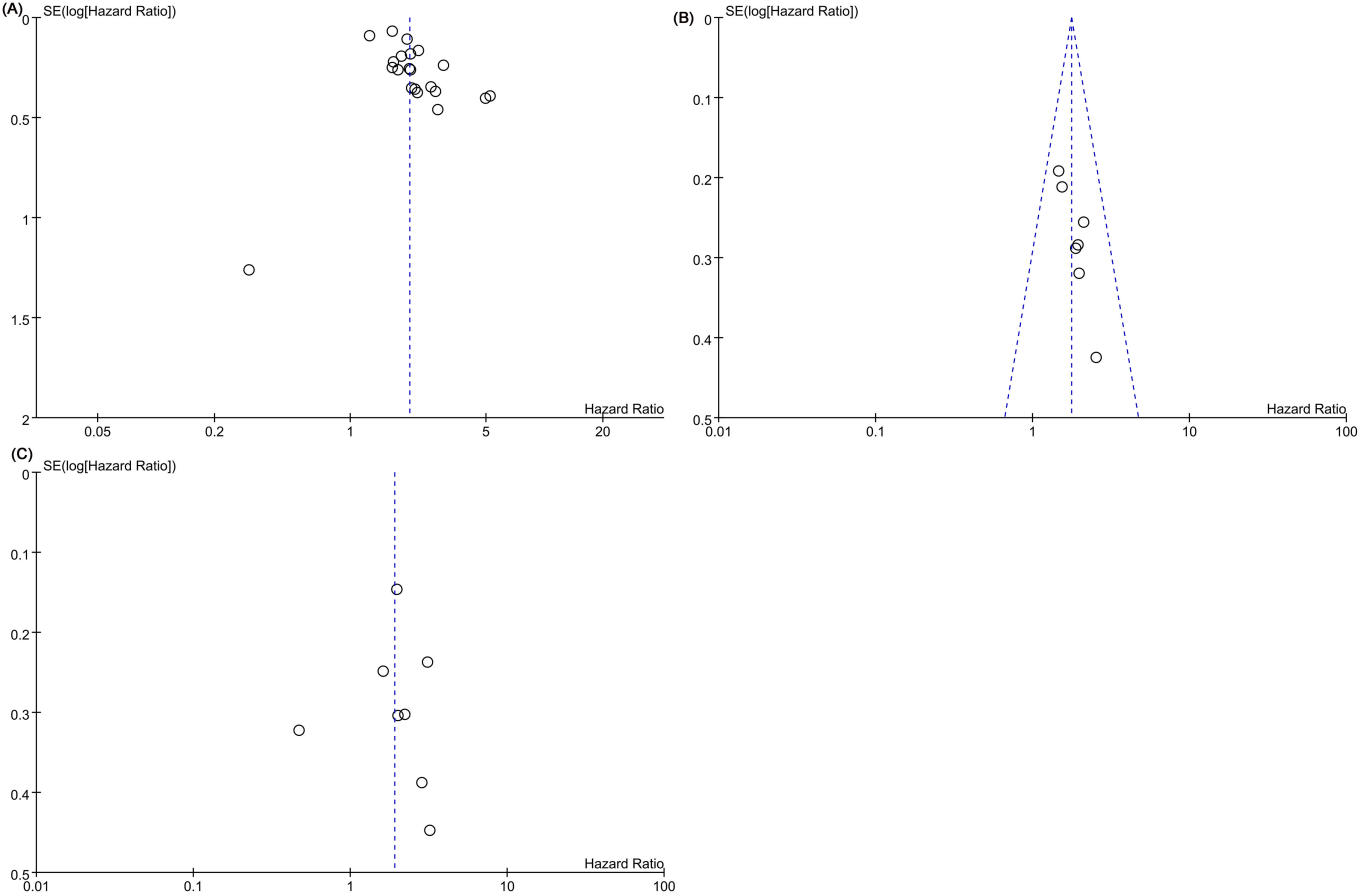
Funnel plots of **(A)** overall survival (OS), **(B)** disease-free survival (DFS), **(C)** recurrence-free survival (RFS).

Publication bias in LCR’s OS prediction was identified through Egger’s test (t=2.93, *P*=0.0009) and funnel plot analysis (**Figure 3A**). Likewise, potential publication bias in LCR’s DFS prediction was observed using Egger’s test (t=4.46, *P*=0.007) and funnel plot (**Figure 3B**). However, no significant publication bias for LCR’s RFS prediction was detected by Egger’s test (t=-0.12, *P*=0.909) and funnel plot analysis (**Figure 3C**).

#### 3.2.5 Sensitivity analysis

**Figure 4:**
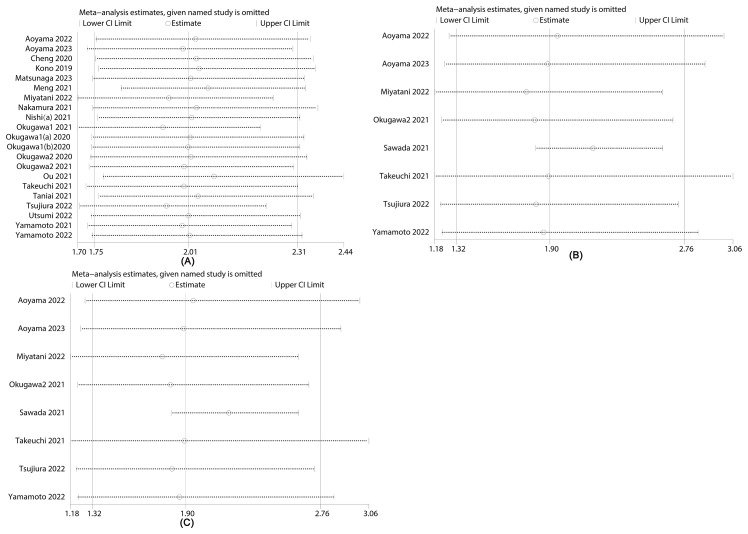
Sensitivity analysis of **(A)** overall survival (OS), **(B)** recurrence-free survival (RFS) and **(C)** disease-free survival (DFS).

**Figure 4** illustrates sensitivity analyses examining the influence of excluding certain literature on overall survival (OS) (**Figure 4A**), recurrence-free survival (RFS) (**Figure 4B**) and disease-free survival (DFS) (**Figure 4C**) significance levels. The results indicate that excluding any literature did not alter the significance of OS, RFS and DFS, underscoring the stability of the relationship between LCR and all outcomes. Notably, upon excluding Sawada 2021, RFS heterogeneity decreased from 73% to 0%. The observed heterogeneity in this study may be attributed to variations in testing methods and statistical approaches. Similarly, heterogeneity in OS could be associated with differences in sample sizes and study designs across populations.

## 4 Discussion

Currently, a growing body of evidence underscores the correlation between low lymphocyte-to-C-reactive protein ratio (LCR) and unfavorable survival outcomes among patients with gastrointestinal cancer. This meta-analysis, incorporating data from 21 studies encompassing 9,131 individuals with gastrointestinal cancer, aims to comprehensively evaluate the prognostic implications of LCR. The findings consistently demonstrate a significant association between diminished LCR levels and reduced overall survival (OS), disease-free survival (DFS), and recurrence-free survival (RFS) in patients with gastrointestinal cancers. Moreover, mounting research emphasizes the impact of systemic inflammation and nutritional status on the long-term prognosis of malignancies, including gastrointestinal cancers (39, 40). Tumor-infiltrating lymphocytes, recognized for their pivotal role in anti-tumor immunity, possess both prognostic and predictive value (41). C-reactive protein (CRP), capable of binding to diverse ligands on damaged cell membranes, strongly activates the classical complement pathway, potentially exacerbating tissue damage and contributing to more severe diseases (42). Elevated CRP levels within the inflammatory and tumor microenvironment promote various cancers (43). Furthermore, serum CRP levels correlate with tumor size, clinicopathological characteristics, and lymph node metastasis (44). Consequently, CRP stands as a crucial biomarker for tumor prognosis and treatment responses (43).

The current meta-analysis reveals that a diminished level of LCR is indicative of a poorer prognosis among patients with gastrointestinal cancer. This aligns with the evidence presented in the encompassed literature, highlighting substantial heterogeneity in OS and RFS. Subgroup analysis identifies cancer type, timing, LCR threshold and treatment method as sources of heterogeneity. Sensitivity analysis reinforces the stability of the meta-analysis, affirming the association between LCR and OS/RFS.

In recent investigations, numerous studies have underscored the superior accuracy of the LCR over other inflammation-related markers in predicting the prognosis of patients afflicted with gastrointestinal cancers. In a comprehensive meta-analysis and systematic review encompassing 2838 patients diagnosed with upper gastrointestinal cancer, Ye et al. elucidated that a diminished the LCR correlates with an unfavorable prognosis in upper gastrointestinal cancer cases (45). Despite these findings, the precise mechanisms underpinning the interplay between the LCR and gastrointestinal cancer prognosis remain elusive. A plausible explanation posited by researchers suggests a potential linkage between the LCR and the immune response among patients with gastrointestinal cancer (46). Lymphocytes consistently feature within the peritumoral inflammatory cell infiltrate, and their presence appears intricately tied to cancer cell activity, hinting at a relationship with tumor growth (47). Furthermore, the association between LCR and preoperative nutritional status, as well as surgical complications, merits attention. Okugawa observed a robust correlation between the LCR and preoperative nutritional status (23). In a study involving 607 patients with gastric cancer, Cheng et al. uncovered a statistically significant disparity in postoperative complications, with an incidence of 20.4% in the low LCR group versus 12.1% in the high LCR group (P = 0.006) (34). These findings underscore a noteworthy correlation between the LCR and postoperative surgical complications. Moreover, Okugawa et al. reported significantly elevated incidences of postoperative infectious complications, surgical site infections, and distant infections in the low LCR group compared to the high LCR group among 477 colorectal cancer patients (postoperative infectious complications: p = 0.0007, surgical site infections: p = 0.01, and distant infections: p = 0.021) (23). A low LCR emerges as an independent risk factor for postoperative complications in colorectal cancer, affirming the substantial association between LCR and postoperative surgical complications. In addition, Yasui et al. (48) found that the LCR and CAR inflammatory markers could accurately predict OS and recurrence‐free survival in patients with stage III CRC, which may identify predictive markers for treatment response and guide personalized therapeutic strategies in patients with gastrointestinal cancer. In conclusion, LCR stands out as the preeminent prognostic indicator among various inflammatory markers, as it is adept at identifying high-risk surgical patients and offering valuable guidance for treatment decisions.

We acknowledge several limitations in the current study. Primarily, the inclusion of mostly retrospective cohort studies introduces potential selection bias. To validate our findings, a prospective, multicenter randomized controlled trial is imperative. Additionally, the exclusively Asian population in our study raises concerns about generalizability; hence, further investigations encompassing diverse regions are warranted to authenticate and extend our discoveries. Moreover, the assessment of heterogeneity using *P*<0.1 and/or *I* ^2^>50% alone may have certain limitations, this is also an unavoidable problem of meta-analysis; therefore, further studies are needed to confirm the findings of this article. Admittedly, The Newcastle–Ottawa Scale (NOS) was used to assess study quality, which is a widely accepted tool. However, the interpretation of NOS scores (seven to nine points indicating high quality) may be subjective. Future studies could provide a more detailed rationale for quality assessment criteria and scoring. Despite these limitations, our study boasts the largest sample size, encompassing all gastrointestinal cancers comprehensively. Our findings affirm that low lymphocyte-to-C-reactive protein ratio (LCR) holds prognostic value, advocating for the development of enhanced prognostic models based on LCR in clinical practice. Although high heterogeneity in overall survival (OS) and recurrence-free survival (RFS) may diminish confidence in our results, additional studies are indispensable to validate our inferences. Furthermore, since existing studies are predominantly retrospective, our study lays a foundation for guiding the design of future large prospective cohort studies. Overall, while the study provides valuable insights into the association between LCR and prognosis in gastrointestinal cancer patients, addressing the highlighted limitations could enhance the validity and generalizability of the findings. Future research could focus on addressing the identified limitations, including incorporating non-English studies, conducting comprehensive assessments of publication bias, exploring alternative approaches for assessing heterogeneity, refining quality assessment criteria, and conducting more extensive sensitivity analyses. Lastly, efforts should be made to translate research findings into clinical practice by conducting prospective validation studies and integrating LCR assessment into routine clinical care pathways. Collaborative efforts between researchers, clinicians, and policymakers are essential to facilitate the translation of biomarker discoveries into clinical utility. Future research could further advance our understanding of the prognostic significance of LCR in gastrointestinal cancer and its potential clinical implications.

## 5 Conclusion

The findings of our investigation indicate that preoperative levels of lymphocyte-to-C-reactive protein ratio (LCR) serve as a valuable adjunctive prognostic marker for gastrointestinal cancer outcomes. Diminished LCR correlates with unfavorable survival rates, enabling identification of high-risk patients in clinical settings. However, heterogeneity exists among the included studies, necessitating further research for result validation.

## Data Availability

All data generated or analyzed during this study are included in this published article and its **Supplementary Information** files.
