# Supplementary material for: The role of lymphocyte-C-reactive protein ratio in the prognosis of gastrointestinal cancer: a systematic review and meta-analysis
Source: Front Oncol. 2024 Aug 29;14:1407306. doi: 10.3389/fonc.2024.1407306 (PMC11390424; doi:10.3389/fonc.2024.1407306)
Supplement: Supplementary file 1 [file Table1.docx]

Supplementary Material

| **Supplementary Table S1** Searching strategy and retrieval formula | |
| --- | --- |
| Searching strategy | ("Lymphocytes"[MeSH Terms] OR ("Lymphocytes"[MeSH Terms] OR "Lymphocytes"[All Fields] OR "lymphocyte"[All Fields] OR "lymphocytic"[All Fields] OR "lymphocyts"[All Fields] OR ("Lymphocytes"[MeSH Terms] OR "Lymphocytes"[All Fields] OR ("lymphoid"[All Fields] AND "cells"[All Fields]) OR "lymphoid cells"[All Fields]) OR ("Lymphocytes"[MeSH Terms] OR "Lymphocytes"[All Fields] OR ("cell"[All Fields] AND "lymphoid"[All Fields]) OR "cell lymphoid"[All Fields]) OR ("Lymphocytes"[MeSH Terms] OR "Lymphocytes"[All Fields] OR ("cells"[All Fields] AND "lymphoid"[All Fields]) OR "cells lymphoid"[All Fields]) OR ("Lymphocytes"[MeSH Terms] OR "Lymphocytes"[All Fields] OR ("lymphoid"[All Fields] AND "cell"[All Fields]) OR "lymphoid cell"[All Fields]))) AND ("C-Reactive Protein"[MeSH Terms] OR ("C-Reactive Protein"[MeSH Terms] OR ("c reactive"[All Fields] AND "protein"[All Fields]) OR "C-Reactive Protein"[All Fields] OR "C-Reactive Protein"[All Fields] OR ("C-Reactive Protein"[MeSH Terms] OR ("c reactive"[All Fields] AND "protein"[All Fields]) OR "C-Reactive Protein"[All Fields] OR "hscrp"[All Fields]) OR ("C-Reactive Protein"[MeSH Terms] OR ("c reactive"[All Fields] AND "protein"[All Fields]) OR "C-Reactive Protein"[All Fields] OR "high sensitivity c reactive protein"[All Fields]) OR ("C-Reactive Protein"[MeSH Terms] OR ("c reactive"[All Fields] AND "protein"[All Fields]) OR "C-Reactive Protein"[All Fields] OR "high sensitivity c reactive protein"[All Fields]) OR ("C-Reactive Protein"[MeSH Terms] OR ("c reactive"[All Fields] AND "protein"[All Fields]) OR "C-Reactive Protein"[All Fields] OR ("hs"[All Fields] AND "crp"[All Fields]) OR "hs crp"[All Fields]))) AND ("ratio"[All Fields] OR "ratio s"[All Fields] OR "ratioes"[All Fields] OR "ratios"[All Fields]) AND ("Gastrointestinal Neoplasms"[MeSH Terms] OR ("Gastrointestinal Neoplasms"[MeSH Terms] OR ("gastrointestinal"[All Fields] AND "neoplasms"[All Fields]) OR "Gastrointestinal Neoplasms"[All Fields] OR ("gastrointestinal"[All Fields] AND "neoplasm"[All Fields]) OR "gastrointestinal neoplasm"[All Fields] OR ("Gastrointestinal Neoplasms"[MeSH Terms] OR ("gastrointestinal"[All Fields] AND "neoplasms"[All Fields]) OR "Gastrointestinal Neoplasms"[All Fields] OR ("neoplasm"[All Fields] AND "gastrointestinal"[All Fields]) OR "neoplasm gastrointestinal"[All Fields]) OR ("Gastrointestinal Neoplasms"[MeSH Terms] OR ("gastrointestinal"[All Fields] AND "neoplasms"[All Fields]) OR "Gastrointestinal Neoplasms"[All Fields] OR ("neoplasms"[All Fields] AND "gastrointestinal"[All Fields]) OR "neoplasms gastrointestinal"[All Fields]) OR ("Gastrointestinal Neoplasms"[MeSH Terms] OR ("gastrointestinal"[All Fields] AND "neoplasms"[All Fields]) OR "Gastrointestinal Neoplasms"[All Fields] OR ("cancer"[All Fields] AND "gastrointestinal"[All Fields] AND "tract"[All Fields]) OR "cancer of gastrointestinal tract"[All Fields]) OR ("Gastrointestinal Neoplasms"[MeSH Terms] OR ("gastrointestinal"[All Fields] AND "neoplasms"[All Fields]) OR "Gastrointestinal Neoplasms"[All Fields] OR ("gastrointestinal"[All Fields] AND "tract"[All Fields] AND "cancer"[All Fields]) OR "gastrointestinal tract cancer"[All Fields]) OR ("Gastrointestinal Neoplasms"[MeSH Terms] OR ("gastrointestinal"[All Fields] AND "neoplasms"[All Fields]) OR "Gastrointestinal Neoplasms"[All Fields] OR ("gastrointestinal"[All Fields] AND "tract"[All Fields] AND "cancers"[All Fields]) OR "gastrointestinal tract cancers"[All Fields]) OR ("Gastrointestinal Neoplasms"[MeSH Terms] OR ("gastrointestinal"[All Fields] AND "neoplasms"[All Fields]) OR "Gastrointestinal Neoplasms"[All Fields] OR ("cancer"[All Fields] AND "gastrointestinal"[All Fields] AND "tract"[All Fields]) OR "cancer of the gastrointestinal tract"[All Fields]) OR ("Gastrointestinal Neoplasms"[MeSH Terms] OR ("gastrointestinal"[All Fields] AND "neoplasms"[All Fields]) OR "Gastrointestinal Neoplasms"[All Fields] OR ("gastrointestinal"[All Fields] AND "cancer"[All Fields]) OR "gastrointestinal cancer"[All Fields]) OR ("Gastrointestinal Neoplasms"[MeSH Terms] OR ("gastrointestinal"[All Fields] AND "neoplasms"[All Fields]) OR "Gastrointestinal Neoplasms"[All Fields] OR ("cancer"[All Fields] AND "gastrointestinal"[All Fields]) OR "cancer gastrointestinal"[All Fields]) OR ("Gastrointestinal Neoplasms"[MeSH Terms] OR ("gastrointestinal"[All Fields] AND "neoplasms"[All Fields]) OR "Gastrointestinal Neoplasms"[All Fields] OR ("cancers"[All Fields] AND "gastrointestinal"[All Fields]) OR "cancers gastrointestinal"[All Fields]) OR ("Gastrointestinal Neoplasms"[MeSH Terms] OR ("gastrointestinal"[All Fields] AND "neoplasms"[All Fields]) OR "Gastrointestinal Neoplasms"[All Fields] OR ("gastrointestinal"[All Fields] AND "cancers"[All Fields]) OR "gastrointestinal cancers"[All Fields])) OR ("Colorectal Neoplasms"[MeSH Terms] OR ("Colorectal Neoplasms"[MeSH Terms] OR ("colorectal"[All Fields] AND "neoplasms"[All Fields]) OR "Colorectal Neoplasms"[All Fields] OR ("colorectal"[All Fields] AND "neoplasm"[All Fields]) OR "colorectal neoplasm"[All Fields] OR ("Colorectal Neoplasms"[MeSH Terms] OR ("colorectal"[All Fields] AND "neoplasms"[All Fields]) OR "Colorectal Neoplasms"[All Fields] OR ("neoplasm"[All Fields] AND "colorectal"[All Fields]) OR "neoplasm colorectal"[All Fields]) OR ("Colorectal Neoplasms"[MeSH Terms] OR ("colorectal"[All Fields] AND "neoplasms"[All Fields]) OR "Colorectal Neoplasms"[All Fields] OR ("neoplasms"[All Fields] AND "colorectal"[All Fields]) OR "neoplasms colorectal"[All Fields]) OR ("colorectal tumours"[All Fields] OR "Colorectal Neoplasms"[MeSH Terms] OR ("colorectal"[All Fields] AND "neoplasms"[All Fields]) OR "Colorectal Neoplasms"[All Fields] OR ("colorectal"[All Fields] AND "tumors"[All Fields]) OR "colorectal tumors"[All Fields]) OR ("colorectal tumour"[All Fields] OR "Colorectal Neoplasms"[MeSH Terms] OR ("colorectal"[All Fields] AND "neoplasms"[All Fields]) OR "Colorectal Neoplasms"[All Fields] OR ("colorectal"[All Fields] AND "tumor"[All Fields]) OR "colorectal tumor"[All Fields]) OR ("Colorectal Neoplasms"[MeSH Terms] OR ("colorectal"[All Fields] AND "neoplasms"[All Fields]) OR "Colorectal Neoplasms"[All Fields] OR ("tumor"[All Fields] AND "colorectal"[All Fields]) OR "tumor colorectal"[All Fields]) OR ("Colorectal Neoplasms"[MeSH Terms] OR ("colorectal"[All Fields] AND "neoplasms"[All Fields]) OR "Colorectal Neoplasms"[All Fields] OR ("tumors"[All Fields] AND "colorectal"[All Fields]) OR "tumors colorectal"[All Fields]) OR ("Colorectal Neoplasms"[MeSH Terms] OR ("colorectal"[All Fields] AND "neoplasms"[All Fields]) OR "Colorectal Neoplasms"[All Fields] OR ("colorectal"[All Fields] AND "cancer"[All Fields]) OR "colorectal cancer"[All Fields]) OR ("Colorectal Neoplasms"[MeSH Terms] OR ("colorectal"[All Fields] AND "neoplasms"[All Fields]) OR "Colorectal Neoplasms"[All Fields] OR ("cancer"[All Fields] AND "colorectal"[All Fields]) OR "cancer colorectal"[All Fields]) OR ("Colorectal Neoplasms"[MeSH Terms] OR ("colorectal"[All Fields] AND "neoplasms"[All Fields]) OR "Colorectal Neoplasms"[All Fields] OR ("cancers"[All Fields] AND "colorectal"[All Fields]) OR "cancers colorectal"[All Fields]) OR ("Colorectal Neoplasms"[MeSH Terms] OR ("colorectal"[All Fields] AND "neoplasms"[All Fields]) OR "Colorectal Neoplasms"[All Fields] OR ("colorectal"[All Fields] AND "cancers"[All Fields]) OR "colorectal cancers"[All Fields]) OR ("Colorectal Neoplasms"[MeSH Terms] OR ("colorectal"[All Fields] AND "neoplasms"[All Fields]) OR "Colorectal Neoplasms"[All Fields] OR ("colorectal"[All Fields] AND "carcinoma"[All Fields]) OR "colorectal carcinoma"[All Fields]) OR ("Colorectal Neoplasms"[MeSH Terms] OR ("colorectal"[All Fields] AND "neoplasms"[All Fields]) OR "Colorectal Neoplasms"[All Fields] OR ("carcinoma"[All Fields] AND "colorectal"[All Fields]) OR "carcinoma colorectal"[All Fields]) OR ("Colorectal Neoplasms"[MeSH Terms] OR ("colorectal"[All Fields] AND "neoplasms"[All Fields]) OR "Colorectal Neoplasms"[All Fields] OR ("carcinomas"[All Fields] AND "colorectal"[All Fields]) OR "carcinomas colorectal"[All Fields]) OR ("Colorectal Neoplasms"[MeSH Terms] OR ("colorectal"[All Fields] AND "neoplasms"[All Fields]) OR "Colorectal Neoplasms"[All Fields] OR ("colorectal"[All Fields] AND "carcinomas"[All Fields]) OR "colorectal carcinomas"[All Fields]))) OR ("Stomach Neoplasms"[MeSH Terms] OR ("Stomach Neoplasms"[MeSH Terms] OR ("stomach"[All Fields] AND "neoplasms"[All Fields]) OR "Stomach Neoplasms"[All Fields] OR ("neoplasm"[All Fields] AND "stomach"[All Fields]) OR "neoplasm stomach"[All Fields] OR ("Stomach Neoplasms"[MeSH Terms] OR ("stomach"[All Fields] AND "neoplasms"[All Fields]) OR "Stomach Neoplasms"[All Fields] OR ("stomach"[All Fields] AND "neoplasm"[All Fields]) OR "stomach neoplasm"[All Fields]) OR ("Stomach Neoplasms"[MeSH Terms] OR ("stomach"[All Fields] AND "neoplasms"[All Fields]) OR "Stomach Neoplasms"[All Fields] OR ("neoplasms"[All Fields] AND "stomach"[All Fields]) OR "neoplasms stomach"[All Fields]) OR ("Stomach Neoplasms"[MeSH Terms] OR ("stomach"[All Fields] AND "neoplasms"[All Fields]) OR "Stomach Neoplasms"[All Fields] OR ("gastric"[All Fields] AND "neoplasms"[All Fields]) OR "gastric neoplasms"[All Fields]) OR ("Stomach Neoplasms"[MeSH Terms] OR ("stomach"[All Fields] AND "neoplasms"[All Fields]) OR "Stomach Neoplasms"[All Fields] OR ("gastric"[All Fields] AND "neoplasm"[All Fields]) OR "gastric neoplasm"[All Fields]) OR ("Stomach Neoplasms"[MeSH Terms] OR ("stomach"[All Fields] AND "neoplasms"[All Fields]) OR "Stomach Neoplasms"[All Fields] OR ("neoplasm"[All Fields] AND "gastric"[All Fields]) OR "neoplasm gastric"[All Fields]) OR ("Stomach Neoplasms"[MeSH Terms] OR ("stomach"[All Fields] AND "neoplasms"[All Fields]) OR "Stomach Neoplasms"[All Fields] OR ("neoplasms"[All Fields] AND "gastric"[All Fields]) OR "neoplasms gastric"[All Fields]) OR ("Stomach Neoplasms"[MeSH Terms] OR ("stomach"[All Fields] AND "neoplasms"[All Fields]) OR "Stomach Neoplasms"[All Fields] OR ("cancer"[All Fields] AND "stomach"[All Fields]) OR "cancer of stomach"[All Fields]) OR ("Stomach Neoplasms"[MeSH Terms] OR ("stomach"[All Fields] AND "neoplasms"[All Fields]) OR "Stomach Neoplasms"[All Fields] OR ("stomach"[All Fields] AND "cancers"[All Fields]) OR "stomach cancers"[All Fields]) OR ("Stomach Neoplasms"[MeSH Terms] OR ("stomach"[All Fields] AND "neoplasms"[All Fields]) OR "Stomach Neoplasms"[All Fields] OR ("gastric"[All Fields] AND "cancer"[All Fields]) OR "gastric cancer"[All Fields]) OR ("Stomach Neoplasms"[MeSH Terms] OR ("stomach"[All Fields] AND "neoplasms"[All Fields]) OR "Stomach Neoplasms"[All Fields] OR ("cancer"[All Fields] AND "gastric"[All Fields]) OR "cancer gastric"[All Fields]) OR ("Stomach Neoplasms"[MeSH Terms] OR ("stomach"[All Fields] AND "neoplasms"[All Fields]) OR "Stomach Neoplasms"[All Fields] OR ("cancers"[All Fields] AND "gastric"[All Fields]) OR "cancers gastric"[All Fields]) OR ("Stomach Neoplasms"[MeSH Terms] OR ("stomach"[All Fields] AND "neoplasms"[All Fields]) OR "Stomach Neoplasms"[All Fields] OR ("gastric"[All Fields] AND "cancers"[All Fields]) OR "gastric cancers"[All Fields]) OR ("Stomach Neoplasms"[MeSH Terms] OR ("stomach"[All Fields] AND "neoplasms"[All Fields]) OR "Stomach Neoplasms"[All Fields] OR ("stomach"[All Fields] AND "cancer"[All Fields]) OR "stomach cancer"[All Fields]) OR ("Stomach Neoplasms"[MeSH Terms] OR ("stomach"[All Fields] AND "neoplasms"[All Fields]) OR "Stomach Neoplasms"[All Fields] OR ("cancer"[All Fields] AND "stomach"[All Fields]) OR "cancer stomach"[All Fields]) OR ("Stomach Neoplasms"[MeSH Terms] OR ("stomach"[All Fields] AND "neoplasms"[All Fields]) OR "Stomach Neoplasms"[All Fields] OR ("cancers"[All Fields] AND "stomach"[All Fields]) OR "cancers stomach"[All Fields]) OR ("Stomach Neoplasms"[MeSH Terms] OR ("stomach"[All Fields] AND "neoplasms"[All Fields]) OR "Stomach Neoplasms"[All Fields] OR ("cancer"[All Fields] AND "stomach"[All Fields]) OR "cancer of the stomach"[All Fields]) OR ("Stomach Neoplasms"[MeSH Terms] OR ("stomach"[All Fields] AND "neoplasms"[All Fields]) OR "Stomach Neoplasms"[All Fields] OR ("gastric"[All Fields] AND "cancer"[All Fields] AND "familial"[All Fields] AND "diffuse"[All Fields])))) OR ("Esophageal Neoplasms"[MeSH Terms] OR ("oesophageal neoplasm"[All Fields] OR "Esophageal Neoplasms"[MeSH Terms] OR ("esophageal"[All Fields] AND "neoplasms"[All Fields]) OR "Esophageal Neoplasms"[All Fields] OR ("esophageal"[All Fields] AND "neoplasm"[All Fields]) OR "esophageal neoplasm"[All Fields] OR ("Esophageal Neoplasms"[MeSH Terms] OR ("esophageal"[All Fields] AND "neoplasms"[All Fields]) OR "Esophageal Neoplasms"[All Fields] OR ("neoplasm"[All Fields] AND "esophageal"[All Fields]) OR "neoplasm esophageal"[All Fields]) OR ("oesophagus neoplasm"[All Fields] OR "Esophageal Neoplasms"[MeSH Terms] OR ("esophageal"[All Fields] AND "neoplasms"[All Fields]) OR "Esophageal Neoplasms"[All Fields] OR ("esophagus"[All Fields] AND "neoplasm"[All Fields]) OR "esophagus neoplasm"[All Fields]) OR ("Esophageal Neoplasms"[MeSH Terms] OR ("esophageal"[All Fields] AND "neoplasms"[All Fields]) OR "Esophageal Neoplasms"[All Fields] OR ("esophagus"[All Fields] AND "neoplasms"[All Fields]) OR "esophagus neoplasms"[All Fields]) OR ("Esophageal Neoplasms"[MeSH Terms] OR ("esophageal"[All Fields] AND "neoplasms"[All Fields]) OR "Esophageal Neoplasms"[All Fields] OR ("neoplasm"[All Fields] AND "esophagus"[All Fields]) OR "neoplasm esophagus"[All Fields]) OR ("Esophageal Neoplasms"[MeSH Terms] OR ("esophageal"[All Fields] AND "neoplasms"[All Fields]) OR "Esophageal Neoplasms"[All Fields] OR ("neoplasms"[All Fields] AND "esophagus"[All Fields]) OR "neoplasms esophagus"[All Fields]) OR ("Esophageal Neoplasms"[MeSH Terms] OR ("esophageal"[All Fields] AND "neoplasms"[All Fields]) OR "Esophageal Neoplasms"[All Fields] OR ("neoplasms"[All Fields] AND "esophageal"[All Fields]) OR "neoplasms esophageal"[All Fields]) OR ("cancer of oesophagus"[All Fields] OR "Esophageal Neoplasms"[MeSH Terms] OR ("esophageal"[All Fields] AND "neoplasms"[All Fields]) OR "Esophageal Neoplasms"[All Fields] OR ("cancer"[All Fields] AND "esophagus"[All Fields]) OR "cancer of esophagus"[All Fields]) OR ("cancer of the oesophagus"[All Fields] OR "Esophageal Neoplasms"[MeSH Terms] OR ("esophageal"[All Fields] AND "neoplasms"[All Fields]) OR "Esophageal Neoplasms"[All Fields] OR ("cancer"[All Fields] AND "esophagus"[All Fields]) OR "cancer of the esophagus"[All Fields]) OR ("oesophagus cancer"[All Fields] OR "Esophageal Neoplasms"[MeSH Terms] OR ("esophageal"[All Fields] AND "neoplasms"[All Fields]) OR "Esophageal Neoplasms"[All Fields] OR ("esophagus"[All Fields] AND "cancer"[All Fields]) OR "esophagus cancer"[All Fields]) OR ("Esophageal Neoplasms"[MeSH Terms] OR ("esophageal"[All Fields] AND "neoplasms"[All Fields]) OR "Esophageal Neoplasms"[All Fields] OR ("cancer"[All Fields] AND "esophagus"[All Fields]) OR "cancer esophagus"[All Fields]) OR ("Esophageal Neoplasms"[MeSH Terms] OR ("esophageal"[All Fields] AND "neoplasms"[All Fields]) OR "Esophageal Neoplasms"[All Fields] OR ("cancers"[All Fields] AND "esophagus"[All Fields]) OR "cancers esophagus"[All Fields]) OR ("oesophagus cancers"[All Fields] OR "Esophageal Neoplasms"[MeSH Terms] OR ("esophageal"[All Fields] AND "neoplasms"[All Fields]) OR "Esophageal Neoplasms"[All Fields] OR ("esophagus"[All Fields] AND "cancers"[All Fields]) OR "esophagus cancers"[All Fields]) OR ("oesophageal cancer"[All Fields] OR "Esophageal Neoplasms"[MeSH Terms] OR ("esophageal"[All Fields] AND "neoplasms"[All Fields]) OR "Esophageal Neoplasms"[All Fields] OR ("esophageal"[All Fields] AND "cancer"[All Fields]) OR "esophageal cancer"[All Fields]) OR ("Esophageal Neoplasms"[MeSH Terms] OR ("esophageal"[All Fields] AND "neoplasms"[All Fields]) OR "Esophageal Neoplasms"[All Fields] OR ("cancer"[All Fields] AND "esophageal"[All Fields]) OR "cancer esophageal"[All Fields]) OR ("Esophageal Neoplasms"[MeSH Terms] OR ("esophageal"[All Fields] AND "neoplasms"[All Fields]) OR "Esophageal Neoplasms"[All Fields] OR ("cancers"[All Fields] AND "esophageal"[All Fields]) OR "cancers esophageal"[All Fields]) OR ("oesophageal cancers"[All Fields] OR "Esophageal Neoplasms"[MeSH Terms] OR ("esophageal"[All Fields] AND "neoplasms"[All Fields]) OR "Esophageal Neoplasms"[All Fields] OR ("esophageal"[All Fields] AND "cancers"[All Fields]) OR "esophageal cancers"[All Fields])))) |
| Retrieval formula | (((("Lymphocytes"[Mesh]) OR (((((Lymphocyte) OR (Lymphoid Cells)) OR (Cell, Lymphoid)) OR (Cells, Lymphoid)) OR (Lymphoid Cell))) AND (("C-Reactive Protein"[Mesh]) OR (((((C Reactive Protein) OR (hsCRP)) OR (High Sensitivity C-Reactive Protein)) OR (High Sensitivity C Reactive Protein)) OR (hs-CRP)))) AND (Ratio)) AND ((((("Gastrointestinal Neoplasms"[Mesh]) OR (((((((((((Gastrointestinal Neoplasm) OR (Neoplasm, Gastrointestinal)) OR (Neoplasms, Gastrointestinal)) OR (Cancer of Gastrointestinal Tract)) OR (Gastrointestinal Tract Cancer)) OR (Gastrointestinal Tract Cancers)) OR (Cancer of the Gastrointestinal Tract)) OR (Gastrointestinal Cancer)) OR (Cancer, Gastrointestinal)) OR (Cancers, Gastrointestinal)) OR (Gastrointestinal Cancers))) OR (("Colorectal Neoplasms"[Mesh]) OR (((((((((((((((Colorectal Neoplasm) OR (Neoplasm, Colorectal)) OR (Neoplasms, Colorectal)) OR (Colorectal Tumors)) OR (Colorectal Tumor)) OR (Tumor, Colorectal)) OR (Tumors, Colorectal)) OR (Colorectal Cancer)) OR (Cancer, Colorectal)) OR (Cancers, Colorectal)) OR (Colorectal Cancers)) OR (Colorectal Carcinoma)) OR (Carcinoma, Colorectal)) OR (Carcinomas, Colorectal)) OR (Colorectal Carcinomas)))) OR (("Stomach Neoplasms"[Mesh]) OR ((((((((((((((((((Neoplasm, Stomach) OR (Stomach Neoplasm)) OR (Neoplasms, Stomach)) OR (Gastric Neoplasms)) OR (Gastric Neoplasm)) OR (Neoplasm, Gastric)) OR (Neoplasms, Gastric)) OR (Cancer of Stomach)) OR (Stomach Cancers)) OR (Gastric Cancer)) OR (Cancer, Gastric)) OR (Cancers, Gastric)) OR (Gastric Cancers)) OR (Stomach Cancer)) OR (Cancer, Stomach)) OR (Cancers, Stomach)) OR (Cancer of the Stomach)) OR (Gastric Cancer, Familial Diffuse)))) OR (("Esophageal Neoplasms"[Mesh]) OR (((((((((((((((((Esophageal Neoplasm) OR (Neoplasm, Esophageal)) OR (Esophagus Neoplasm)) OR (Esophagus Neoplasms)) OR (Neoplasm, Esophagus)) OR (Neoplasms, Esophagus)) OR (Neoplasms, Esophageal)) OR (Cancer of Esophagus)) OR (Cancer of the Esophagus)) OR (Esophagus Cancer)) OR (Cancer, Esophagus)) OR (Cancers, Esophagus)) OR (Esophagus Cancers)) OR (Esophageal Cancer)) OR (Cancer, Esophageal)) OR (Cancers, Esophageal)) OR (Esophageal Cancers)))) |

| **Supplementary Table S2** Quality evaluation of the eligible studies with Newcastle–Ottawa scale. | | | | | | | | | |
| --- | --- | --- | --- | --- | --- | --- | --- | --- | --- |
| Study | Selection | | | | Comparability | | Outcome | | |
|  | Representative-ness | Selection of  non-exposed | Ascertainment  of exposure | Outcome not present at start | Comparability on most important factors | Comparability on other risk factors | Assessment of outcome | Long enough follow-up (median≥1 year) | Adequacy  (completeness) of follow-up |
| Aoyama et al.([13](#_ENREF_9)) | * | * | * | * | * | * | * | * | * |
| Yamamoto et al.([12](#_ENREF_10)) | * | * | * | * | - | - | * | * | * |
| Aoyama et al.([11](#_ENREF_11)) | * | * | * | * | - | - | * | * | * |
| Yamamoto et al.([10](#_ENREF_12)) | * | * | * | * | * | * | * | * | * |
| Tsujiura et al.([13](#_ENREF_13)) | * | * | * | * | * | * | * | - | * |
| Miyatani et al.([14](#_ENREF_14)) | * | * | * | * | * | * | * | * | * |
| Okugawa et al. ([14](#_ENREF_29)) | * | * | * | * | * | * | * | * | * |
| Nishi et al.([11](#_ENREF_19)) | * | * | * | * | * | * | * | * | * |
| Okugawa et al.([21](#_ENREF_20)) | * | * | * | * | * | - | * | * | * |
| Okugawa et al.([15](#_ENREF_22)) | * | * | * | * | * | - | * | * | * |
| Takeuchi et al.([10](#_ENREF_23)) | * | * | * | * | * | * | * | * | * |
| Nakamura et al.([8](#_ENREF_24)) | * | * | * | * | * | * | * | * | * |
| Utsumi et al.([11](#_ENREF_25)) | * | * | * | * | * | * | * | * | * |
| Kono et al.([11](#_ENREF_26)) | * | * | * | * | * | - | * | * | * |
| Matsunaga et al.([13](#_ENREF_28)) | * | * | * | * | * | * | * | * | * |
| Okugawa et al.([14](#_ENREF_29)) | * | * | * | * | * | * | * | * | * |
| Meng et al.(7) | * | * | * | * | * | - | * | * | * |
| Taniai et al.(8) | * | * | * | * | * | * | * | * | * |
| Ou et al.(5) | * | * | * | * | * | - | * | * | * |
| Cheng et al.(4) | * | * | * | * | * | * | * | * | * |
| Sawada et al.(11) | * | * | * | * | * | * | * | * | * |
| *indicates criterion met; - indicates significant of criterion not met. | | | | | | | | | |

1.Aoyama T, Nakazano M, Nagasawa S, Hara K, Komori K, Tamagawa H, et al.

The association of the lymphocyte-to-C-reactive-protein ratio with gastric cancer

patients who receive curative treatment. In Vivo (Athens Greece). (2022) 36:482–9.

doi: 10.21873/invivo.12728

2. Yamamoto T, Fukuda M, Okuchi Y, Oshimo Y, Nishikawa Y, Hisano K, et al.

Clinical impact of lymphocyte/C-reactive protein ratio on postoperative outcomes in

patients with rectal cancer who underwent curative resection. Sci Rep. (2022) 12:17136.

doi: 10.1038/s41598-022-21650-1

3.Aoyama T, Nagasawa S, Nakazono M, Segami K, Tamagawa H, Tamagawa A,

et al. The clinical impacts of lymphocyte-to-C-reactive protein ratio for esophageal

cancer patients who receive curative treatment. J Cancer Res Ther. (2023) 19:556–61.

doi: 10.4103/jcrt.jcrt_139_21

4. Yamamoto A, Toiyama Y, Okugawa Y, Ichikawa T, Imaoka H, Yasuda H, et al.

Clinical implications of the preoperative lymphocyte C-reactive protein ratio in

esophageal cancer patients. Surg Today. (2021) 51:745–55. doi: 10.1007/s00595-020-

02166-5

5. Tsujiura M, Yamamoto A, Imaoka H, Shimura T, Kitajima T, Morimoto Y, et al.

Clinical utility of lymphocyte to C-reactive protein ratio in predicting survival and

postoperative complication for esophago-gastric junction cancer. Surg Oncol. (2022)

44:101842. doi: 10.1016/j.suronc.2022.101842

6. Miyatani K, Sawata S, Makinoya M, Miyauchi W, Shimizu S, Shishido Y, et al.

Combined analysis of preoperative and postoperative lymphocyte-C-reactive protein

ratio precisely predicts outcomes of patients with gastric cancer. BMC cancer. (2022)

22:641. doi: 10.1186/s12885-022-09716-9

7. Okugawa Y, Fujikawa H, Omura Y, Yamamoto A, Kitajima T, Shimura T, et al.

Cumurative periopertive lymphocyte/C-reactive protein ratio as a predictor of longterm

outcomes in patients with colorectal cancer. Gastroenterology. (2021) 160:S–887.

doi: 10.1016/S0016-5085(21)02849-3

8. Nishi M, Shimada M, Tokunaga T, Higashijima J, Yoshikawa K, Kashihara H,

et al. Lymphocyte to C-reactive protein ratio predicts long-term outcomes for patients

with lower rectal cancer. World J Surg Oncol. (2021) 19:201. doi: 10.1186/s12957-021-

02319-x

9. Okugawa Y, Toiyama Y, Yamamoto A, Shigemori T, Ide S, Kitajima T, et al.

Lymphocyte-C-reactive protein ratio as promising new marker for predicting surgical

and oncological outcomes in colorectal cancer. Ann surge. (2020) 272:342–51.

doi: 10.1097/SLA.0000000000003239

10. Okugawa Y, Toiyama Y, Yamamoto A, Shigemori T, Ichikawa T, Yin C, et al.

Lymphocyte-to-C-reactive protein ratio and score are clinically feasible nutritioninflammation

markers of outcome in patients with gastric cancer. Clin Nutr (Edinburgh

Scotland). (2020) 39:1209–17. doi: 10.1016/j.clnu.2019.05.009

11. Takeuchi M, Kawakubo H, Hoshino S, Matsuda S, Mayanagi S, Irino T, et al.

Lymphocyte-to-C-reactive protein ratio as a novel marker for predicting oncological

outcomes in patients with esophageal cancer. World J surge. (2021) 45:3370–7.

doi: 10.1007/s00268-021-06269-z

12. Nakamura Y, Shida D, Boku N, Yoshida T, Tanabe T, Takamizawa Y, et al.

Lymphocyte-to-C-reactive protein ratio is the most sensitive inflammation-based

prognostic score in patients with unresectable metastatic colorectal cancer. Dis colon

rectum. (2021) 64:1331–41. doi: 10.1097/DCR.0000000000002059

13. Utsumi M, Inagaki M, Kitada K, Tokunaga N, Kondo M, Yunoki K, et al.

Lymphocyte-to-C-reactive protein ratio predicts prognosis in patients with colorectal

liver metastases post-hepatic resection: A retrospective study. Anticancer Res. (2022)

42:4963–71. doi: 10.21873/anticanres.16003

14. Kono Y, Saito H, Murakami Y, Shishido Y, Kuroda H, Matsunaga T, et al.

Postoperative ratio of the maximum C-reactive protein level to the minimum

peripheral lymphocyte count as a prognostic indicator for gastric cancer patients.

Surg Today. (2019) 49:206–13. doi: 10.1007/s00595-018-1724-x

15. Matsunaga T, Saito H, Fukumoto Y, Kuroda H, Taniguchi K, Takahashi S, et al.

The prognostic impact of the lymphocyte-to-C-reactive protein ratio in patients with

unresectable or recurrent advanced gastric cancer treated with first- and second-line

treatment. Surg Today. (2023) 53:940–8. doi: 10.1007/s00595-022-02638-w

16. Okugawa Y, Toiyama Y, Fujikawa H, Ide S, Yamamoto A, Omura Y, et al.

Prognostic potential of lymphocyte-C-reactive protein ratio in patients with rectal

cancer receiving preoperative chemoradiotherapy. J gastrointest Surg. (2021) 25:492–

502. doi: 10.1007/s11605-019-04495-4

17.Meng Y, Long C, Huang X, Huang L, Liao L, Tang W, et al. Prognostic role and

clinical significance of C-reactive protein-lymphocyte ratio in colorectal cancer.

Bioengineered. (2021) 12:5138–48. doi: 10.1080/21655979.2021.1960768

18. Taniai T, Haruki K, Hamura R, Fujiwara Y, Furukawa K, Gocho T, et al. The

prognostic significance of C-reactive protein-to-lymphocyte ratio in colorectal liver

metastases. J Surg Res. (2021) 258:414–21. doi: 10.1016/j.jss.2020.08.059

19. Ou W, Zhou C, Zhu X, Lin L, Xu Q. Prognostic significance of preoperative

lymphocyte-to-C-reactive protein ratio in patients with non-metastatic colorectal

cancer. OncoTargets Ther. (2021) 14:337–46. doi: 10.2147/OTT.S290234

20. Cheng CB, Zhang QX, Zhuang LP, Sun JW. Prognostic value of lymphocyte-to-

C-reactive protein ratio in patients with gastric cancer after surgery: a multicentre

study. Japanese J Clin Oncol. (2020) 50:1141–9. doi: 10.1093/jjco/hyaa099

21. Sawada R, Akiyoshi T, Kitagawa Y, Hiyoshi Y, Mukai T, Nagasaki T, et al.

Systemic inflammatory markers combined with tumor-infiltrating lymphocyte density

for the improved prediction of response to neoadjuvant chemoradiotherapy in rectal

cancer. Ann Surg Oncol. (2021) 28:6189–98. doi: 10.1245/s10434-021-09975-z
